# *Arabidopsis thaliana* DGAT3 is a [2Fe-2S] protein involved in TAG biosynthesis

**DOI:** 10.1038/s41598-018-35545-7

**Published:** 2018-11-22

**Authors:** Laure Aymé, Simon Arragain, Michel Canonge, Sébastien Baud, Nadia Touati, Ornella Bimai, Franjo Jagic, Christelle Louis-Mondésir, Pierre Briozzo, Marc Fontecave, Thierry Chardot

**Affiliations:** 1 Institut Jean-Pierre Bourgin, INRA, AgroParisTech, CNRS, Université Paris-Saclay, 78000 Versailles, France; 2 Laboratoire de Chimie des Processus Biologiques, UMR 8229 CNRS, Collège de France, Université Paris 6, 11 Place Marcelin Berthelot, 75231 Paris, CEDEX 05, France; 3 Chimie ParisTech, PSL Research University, CNRS, Institut de Recherche de Chimie Paris (IRCP), F-75005 Paris, France

## Abstract

Acyl-CoA:diacylglycerol acyltransferases 3 (DGAT3) are described as plant cytosolic enzymes synthesizing triacylglycerol. Their protein sequences exhibit a thioredoxin-like ferredoxin domain typical of a class of ferredoxins harboring a [2Fe-2S] cluster. The *Arabidopsis thaliana* DGAT3 (AtDGAT3; At1g48300) protein is detected in germinating seeds. The recombinant purified protein produced from *Escherichia coli*, although very unstable, exhibits DGAT activity *in vitro*. A shorter protein version devoid of its N-terminal putative chloroplast transit peptide, Δ46AtDGAT3, was more stable *in vitro*, allowing biochemical and spectroscopic characterization. The results obtained demonstrate the presence of a [2Fe-2S] cluster in the protein. To date, AtDGAT3 is the first metalloprotein described as a DGAT.

## Introduction

Triacylglycerols (TAGs) are highly reduced and anhydrous storage compounds representing a widespread source of energy and carbon store in eukaryotes. Cells store TAGs in dedicated organelles called lipid droplets (LDs). LDs comprise a hydrophobic core of neutral lipids, TAGs and steryl esters (SEs), surrounded by a monolayer of phospholipids (PLs) which contains various types of specialized proteins^1^. In eukaryotes, three different types of enzymes can synthesize TAG from *sn*-1,2-diacylglycerol (DAG). Phospholipid: diacylglycerol acyltransferase (PDAT, EC 2.3.1.158) uses PL as acyl donor^2,3^. DAG transacylase (EC 2.3.1.124) transfers an acyl group from one DAG to another. This activity has been reported in various organisms^4,5^ although corresponding coding sequences remain to be identified. Acyl-CoA:diacylglycerol acyltransferases (DGATs, EC 2.3.1.20) are acyl-CoA dependent enzymes which catalyze the last and only committed step of the Kennedy pathway^6^.

Eukaryotic DGATs are classified in three distinct classes. DGAT1 and 2 types are integral membrane proteins of the endoplasmic reticulum^7–10^ whereas DGAT3 are soluble proteins^11–13^. They share no sequence homology and probably result from convergent evolution^6,14^. Members of DGAT1 and DGAT2 families participate in TAG synthesis in mammals^15,16^, plants^9,17,18^ and yeasts^19–21^, and presumably have different physiological functions. In *A. thaliana*, AtDGAT1 makes a major contribution to seed oil content^18,22,23^. In several crops producing non-edible oils, members of the DGAT2 family incorporate unusual fatty acids (FA) into seed TAGs^9,24,25^. Soluble DGATs were identified in plants^26^. *Arachis hypogea* DGAT3-1 (AhDGAT3-1) was purified from the cytosolic fraction of developing peanut cotyledons^13^. Two other isoforms were also identified in peanut: AhDGAT3-2 and AhDGAT3-3. The latter was functionally characterized by heterologous expression in yeast^11^.

Induction of *AtDGAT3* was observed 12 hours after imbibition in the *pxa1* mutant disrupted for the PEROXISOMAL ABC TRANSPORTER 1 (PXA1) gene involved in the transport of fatty acids. Five days after imbibition, *pxa1* seedlings grown on sugar exhibited a significant enrichment in TAG species containing C18:3 and, to a lesser extent, C18:2 acyl chains in comparison with wild-type lines. AtDGAT3 involvement in TAG remodeling as well as its specificity toward C18:3 and C18:2 acyl chains was confirmed by transient expression in leaves of *Nicotiana benthamiana*^12^. Transient expression of AtDGAT3 in this system also increased the leaf TAG content. *AtDGAT3* is highly and ubiquitously expressed in *A. thaliana* and could therefore fulfill housekeeping function(s), such as the regulation of acyl exchanges between TAGs and the cytosolic acyl-CoA pool^12^. Other soluble plant acyltransferases have been identified over the past decades^27–30^. Altogether, these enzymes could create a cytosolic route of TAG synthesis. Nevertheless, the DGAT activity of AtDGAT3 still needs to be validated *in vitro* with the purified protein, or in *vivo* by functional complementation of a mutant defective in TAG synthesis.

Here we report the biochemical and functional characterization of AtDGAT3. The enzyme was detected by Western blot in germinating seeds of *A. thaliana*. *In vitro*, we demonstrated that AtDGAT3 was active. An *in silico* analysis of the protein sequence revealed the presence of a thioredoxin-like ferredoxin domain harboring four conserved cysteines possibly involved in the binding of a [2Fe-2S] cluster. While purifying in aerobic conditions the bacterially expressed protein, we observed a brown color associated with AtDGAT3. Spectroscopic characterization of the purified protein and iron and sulfur quantification established that AtDGAT3 is an iron-sulfur protein. Nevertheless, the presence of a thioredoxin-like ferredoxin domain, intriguing for a predicted DGAT, does not convey precise information on the activity of the protein^31^ and raises questions about its possible biochemical function.

## Materials and Methods

### Amplification of *AtDGAT3* transcripts *in planta*

*AtDGAT3* transcripts were amplified from a mixture of cDNA prepared from maturing seeds aged 14 days after flowering, using Phusion High-Fidelity DNA Polymerase (Thermo Fisher Scientific, Illkirch, France). PCR products were sequenced (GATC biotech, Cologne, Germany).

### Identification of the expressed form of *AtDGAT3* protein *in planta*

*A. thaliana* seeds (Col-0 accession) were incubated on humid Whatman discs at 4 °C in the dark for 72 hours and then transferred in a growth cabinet (25 °C, in constant light). Proteins were extracted from seeds after various incubation times using a Fast Prep Homogenizer (MP Biomedical, Illkirch, France) in NuPAGE LDS sample buffer (ThermoFisher Scientific) containing 100 mM DTT. Western blotting was performed as described below in the SDS-PAGE and immunoblotting section.

### Cloning of *AtDGAT3* for expression in *E. coli*

Three AtDGAT3 variants, differing by their N-terminal extension, were expressed: (i) the full-length 360-residues AtDGAT3 corresponding to the predicted sequence available on the TAIR website from 2017-07-24, (ii) a truncated Δ46AtDGAT3 protein lacking the putative chloroplast transit peptide predicted by the ChloroP 1.1 Server (http://www.cbs.dtu.dk/services/ChloroP/) and (iii) the Δ75AtDGAT3 truncated form corresponding to the predicted AtDGAT3 coding sequence displayed on the TAIR website until 2017-07-23.

Expression plasmid containing AtDGAT3 sequence was a generous gift from Prof. Ivo Feussner. *Δ46AtDGAT3* coding sequence was amplified from the above-mentioned plasmid by PCR using Phusion High-Fidelity DNA Polymerase. The forward primer (5′ GGG**CATATG**TGCAACAA 3′) containing a NdeI restriction site (bold), and the reverse primer (5′ GGGG**CTCGAG**ATATGAGACAGAACCGAGTCC 3′) containing a XhoI restriction site (bold) were designed from At1g48300 sequence. *Δ75AtDGAT3* coding sequence was amplified by PCR using Phusion High-Fidelity DNA Polymerase and the *A. thaliana* cDNA as template (*E. coli* C00232(E) clone; GenBank Accession number AF344322; Arabidopsis Biological Resource Center). The forward primer used (5′ GGGGGGG**CATATG**GAGAAGGAGAAGAAGGC 3′) contained a NdeI restriction site (bold), and the reverse primer (5′ GGGG**CTCGAG**ATATGAGACAGAACCGAGTCC 3′) contained a XhoI restriction site (bold).

The amplified PCR fragments were inserted into the multiple cloning site of the pET-32b (+) vector (Millipore S.A.S., Molsheim, France), in frame with a sequence coding a 6xHis tag at the protein C-terminal. The constructs were sequenced to confirm the absence of mutation. According to their residue contents, the calculated molecular mass of the His-tagged recombinant proteins is 40.2, 35.4 and 31.9 kDa for the three different forms, respectively (ProtParam tool on the ExPASy portal: http://web.expasy.org/protparam/).

### Bacterial expression of recombinant AtDGAT3

Plasmid constructs were used to transform *Escherichia coli* T7 Express I^q^ competent cells (New England BioLabs, Ipswich, USA). Cells were grown in Luria-Bertani medium at 37 °C and 250 rpm until the OD_600_ reached 0.8. Temperature was lowered to 18 °C, then 1 mM isopropyl-β-d-thiogalactopyranoside (IPTG) was added to induce protein expression. Cells were grown overnight. Cells were harvested by centrifugation at 6000 × *g* for 15 min and washed with NaCl 0.9% (*w*/*v*). Cell pellets (6000 x g for 10 min) were snap frozen in liquid nitrogen and stored at −80 °C.

### Purification of bacterially expressed 6xHis-tagged AtDGAT3

All purification steps were performed at 4 °C. Bacterial cell pellets were thawed and resuspended in lysis buffer (100 mM triethanolamine pH 7.5, 300 mM NaCl, 5% (*v*/*v*) glycerol, 0.5 mM tris(2-carboxyethyl)phosphine (TCEP)) containing protease inhibitor (cOmplete Mini EDTA-free, Roche, Indianapolis, USA). Cells were disrupted with a One Shot cell disruptor (Constant Systems Ltd, UK) at a pressure of 1.96 kbar. Extract was spun at 12 000 × *g* for 10 min at 4 °C. The supernatant was supplemented with 0.25% (*w*/*v*) *N*-lauroylsarcosine (Sigma-Aldrich) and loaded onto a HisTrap HP column connected to an ÄKTA purifier system (GE Healthcare Life Sciences, Vélizy-Villacoublay, France) and pre-equilibrated with the lysis buffer supplemented with 0.25% (*w*/*v*) *N*-lauroylsarcosine. Unbound proteins were washed using the lysis buffer supplemented with 0.25% (*w*/*v*) *N*-lauroylsarcosine and bound proteins were eluted with a linear gradient of 0–400 mM imidazole (Sigma-Aldrich). Purified proteins were concentrated using an Amicon Ultra-15 device (Merck Millipore, Darmstadt, Germany) with a molecular weight cut-off of 30 kDa, and separated by size-exclusion chromatography (SEC). The concentrated proteins were loaded onto a Superdex 200 10/300 GL column connected to an ÄKTA purifier system and pre-equilibrated with the gel-filtration buffer (100 mM Triethanolamine pH 7.5, 300 mM NaCl, 5% Glycerol, 1% n-Octyl-β-D-glucopyranoside, 1 mM TCEP). Elution was performed using an isocratic flux of buffer. After SEC, the purified protein was snap frozen in liquid nitrogen and stored at −80 °C. Column was calibrated with gel filtration standard (Bio-Rad, Marnes-la-Coquette, France).

### SDS-PAGE and immunoblotting

Proteins were separated using 10% Bis-Tris NuPAGE gels with MOPS SDS running buffer and NuPAGE LDS sample buffer (ThermoFisher Scientific) containing 50 mM DTT according to the manufacturer’s recommendations. The gels were either stained with Coomassie blue G-250 according to^32^ or transferred to a PVDF membrane (Immobilon-P, 0.45 mm pore size, Merck Millipore). AtDGAT3 was detected using polyclonal antibodies raised in rats (Agro-Bio, La Ferté Saint Aubin, France) against a purified bacterially expressed truncated Δ75 form of AtDGAT3 (according to the gene sequence available on TAIR until 2017-07-24). Secondary antibody was a horseradish peroxidase-conjugated anti-rat IgG (Santa Cruz Biotechnology, Heidelberg, Germany).

### Proteomics analysis of the purified protein

The purified recombinant protein was separated by SDS-PAGE and bands were excised from the gel. In-gel tryptic digestion was performed with the Progest system (Genomic Solution) according to^33^ after protein reduction (10 mM DTT) and alkylation (55 mM iodoacetamide). NanoLC-MS/MS analysis was performed using an Ultimate 3000 LC system (Dionex, Sunnyvale, CA) connected to a LTQ Orbitrap mass spectrometer (Thermo Electron, Waltham, MA) according to^34^. Database searches were performed using X!Tandem Pipeline version 3.3.3 according to^35^. Identifications of peptides were performed using the UniProt and the TAIR databases. Data were filtered according to a peptide E-value smaller than 0.05 with a minimum of two peptides to identify a protein.

### Concentration measurements

Protein concentration was determined using Bio-Rad Protein Assay (Bio-Rad, Marnes-la-Coquette, France) with bovine serum albumin as a standard.

Iron and sulfur in purified protein samples were respectively quantified by Fish^36^ and Beinert^37^ colorimetric methods using a Fe^2+^ standard solution (Sigma-Aldrich) or a standard solution obtained from Na_2_S.9H_2_O crystals (Sigma-Aldrich).

### AtDGAT3 spectroscopies

UV-Visible absorption spectra were recorded in quartz cuvettes using a UV-1800 Shimadzu spectrophotometer.

For electron paramagnetic resonance (EPR) analysis, two solutions were analyzed (i) without the addition of sodium dithionite, (ii) with 1 mM sodium dithionite incubated 10 min with Δ46AtDGAT3 sample, using a freshly prepared stock of 100 mM sodium dithionite (Sigma-Aldrich) kept in a MBraun glovebox (O_2_ < 0.5 ppm). Each solution was introduced into EPR quartz tubes. A standard Cu-EDTA solution was used (200 µM) in 25 mM Tris-HCl pH 8, 200 mM NaCl, in order to quantify AtDGAT3 signal. Continuous wave EPR experiments were performed on a X-Band ELEXSYS E500 spectrometer (Bruker BioSpin S.A.S., Wissembourg, France) operating at 9.39 GHZ and equipped with ST cavity cooled by an helium flow cryostat ESR 900 (Oxford Instruments, Austin, USA). The continuous wave EPR spectra of frozen solution were recorded at 50 K under non-saturating conditions and using the following parameters: a microwave power of 10 mW, a modulation amplitude of 5 G, a receiver gain of 40 dB and an accumulation of 10 scans.

### DGAT assay

The diacylglycerol acyltransferase activity assay used was modified from Sanderson *et al*.^38^ and performed as described by Haili *et al*.^39^ using a reaction mixture containing 60 µg of the purified enzyme in 100 mM phosphate buffer pH 8 and 100 µM 1,2-Dioleoyl-*sn*-glycerol (Cayman chemical, Ann Arbor, USA) in a final volume of 100 µl. The reaction mixture was incubated with 50 µM linoleoyl-CoA (Sigma-Aldrich). The Dga1p∆19 protein from *Y. lipolytica*, used as positive control, was obtained according to Haili *et al*.^39^. Substrates and products were separated on HPTLC plates and lipids visualized using methanolic cupric sulfate oxidation according to the same authors^39^.

## Results

### AtDGAT3 contains a thioredoxin-like ferredoxin domain conserved among members of the DGAT3 family

Recently, the predicted structure of the *AtDGAT3* (At1g48300) gene has been revised (TAIR website, www.arabidopsis.org). Previous annotations and reports^12^ described an 858-bp coding sequence (CDS). A revised annotation proposed a new translational start site for *AtDGAT3* yielding a longer CDS comprising 1,083 bp. Using a RT-PCR approach, we could amplify and sequence the corresponding CDS from a mixture of cDNA prepared from *A. thaliana* seeds. The protein encoded by this new CDS comprises 360 residues.

**Figure 1:**
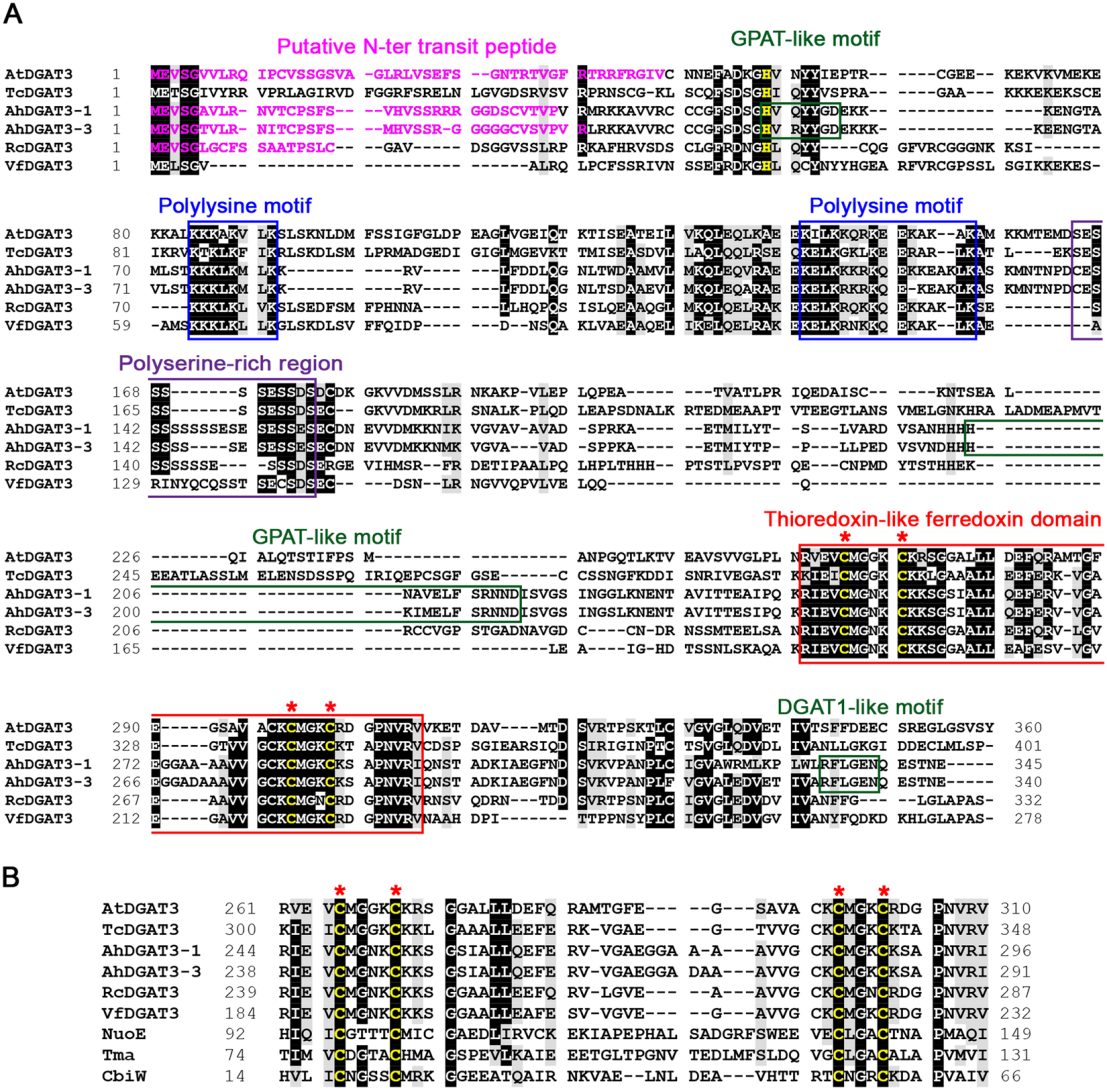
Comparison of AtDGAT3 sequence with that of other DGAT3s. The AtDGAT3 amino acid sequence (GenBank accession number AEE32272) was aligned, using Clustal Omega with (**A**) five plant DGAT3 protein sequences [*Theobroma cacao* (EOX95446), *Arachis hypogaea* (AAX62735 and AGT57760), *Ricinus communis* (EEF43203), *Vernicia fordii* (AGL81309)] and with (**B**) homologues to the thioredoxin-like [2Fe-2S] ferredoxin class: a ferredoxin from *Bacillus megaterium* (CbiW, CAA04306), a NADH-ubiquinone oxidoreductase subunit from *Paracoccus denitrificans* (NuoE or NQO2, AAA25588), the [FeFe]-hydrogenase gamma subunit from *Thermotoga maritima* (Tma or HydC, AAC02684)]. Multiple sequence alignments were edited using the BioEdit program and the BLOSUM62 similarity matrix for shading with a threshold of 75%. Identical residues are highlighted in black and homologous residues are shaded in grey. Conserved motifs are boxed: the two polylysine motifs in blue, a polyserine-rich region in purple and the thioredoxin-like ferredoxin domain in red with its conserved cysteine residues in yellow and highlighted with red asterisks. Putative catalytic motifs identified in *A. hypogea* DGAT3s by homology with motifs found in GPATs or DGAT1s are boxed in green. The putative catalytic histidine from the first GPAT-like motif, absent in the truncated forms of AtDGAT3, is yellow.

AtDGAT3 only shares 28% sequence identity with *Arachis hypogea* AhDGAT3-1, the first characterized member of the DGAT3 family^13^. DGAT3 protein sequences are moderately conserved with only 49 identical residues among the six plant DGAT3 sequences aligned in Fig. 1A. A chloroplast transit peptide located at the N-terminal extremity of the protein is predicted by ChloroP 1.1 (http://www.cbs.dtu.dk/services/ChloroP/) for *A. thaliana*, *A. hypogaea*, and *R. communis* DGAT3s, but not for *T. cacao* and *V. fordii*’s proteins. Noteworthy, this predicted transit peptide was absent in the shorter Δ75AtDGAT3 protein version that was used by Hernandez and colleagues to study the subcellular localization of AtDGAT3^12^.

The conserved residues are scattered in the N-terminal and C-terminal regions of AtDGAT3s. Two polylysine motifs are found in the first half of DGAT3 proteins (residues 84–91 and 141–155 of AtDGAT3). The C-terminal region (residues 261–310 of AtDGAT3 in Fig. 1B) is the most conserved among DGAT3 proteins. It harbors a domain typical of the thioredoxin-like [2Fe-2S] ferredoxin family (cd02980 from NCBI’s conserved domain database,^40^) with features only found in homologues to this class of ferredoxins. These homologues exhibit various activities: one is a subunit of a NADH-ubiquinone oxidoreductase from mitochondrial and bacterial respiratory chains, another one is a hydrogenase subunit^31^. They all harbor 4 strictly conserved cysteines within a particular motif also found in plant DGAT3s (C-X_4_-C-X_27–34_-C-X_3_-C). Conserved cysteines are involved in the binding of a [2Fe-2S] cluster in members of the thioredoxin-like ferredoxin family^41,42^.

### AtDGAT3 protein is expressed in germinating seeds

**Figure 2:**
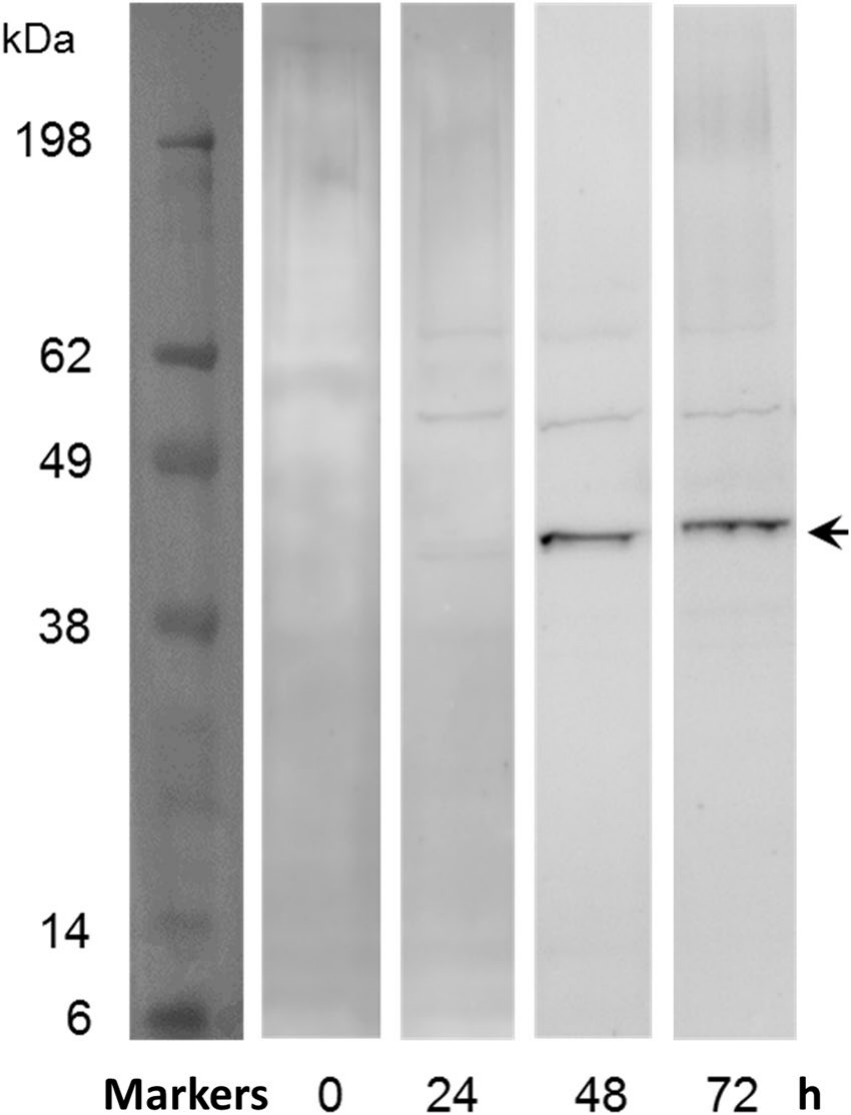
Immunodetection of AtDGAT3 in *A. thaliana* in germinating seeds. *A. thaliana* seeds (from the Col-0 accession) were stratified on humid Whatman discs at 4 °C in the dark for 72 hours and then transferred in a growth cabinet (25 °C, constant light) to trigger germination. Proteins were extracted from 10 seeds or seedlings during the germination process (0, 24, 48, or 72 h after the first exposure to the light), separated by SDS-PAGE, transferred and immunodetected using polyclonal antibodies raised in rat against Δ75AtDGAT3. The arrow indicates the localization of the protein (calculated MW = 39.2 kDa). Separations indicate delineation of the figure.

We used antibodies raised against a truncated form of AtDGAT3 (lacking 75 residues at the N-terminus) for detecting protein accumulation in seeds upon imbibition. Imbibed seeds were stratified for 72 h in the dark at 4 °C to release residual dormancy, and then transferred in a growth cabinet (continuous light, 25 °C) to trigger germination. Germinating seeds were harvested every 24 h upon first exposure to the light. After 48 h of light exposure, immunoreactive material was detected by Western blot (Fig. 2) in germinating seedlings. The molecular weight of the bands observed slightly increased with time. As a positive control, we used the purified recombinant AtDGAT3, indicating the position of the full-length enzyme (arrow). The presence of AtDGAT3 in germinated seeds was confirmed by proteomics as deduced from the identification of at least 2 different peptides from the protein.

### Purification of recombinant AtDGAT3

**Figure 3:**
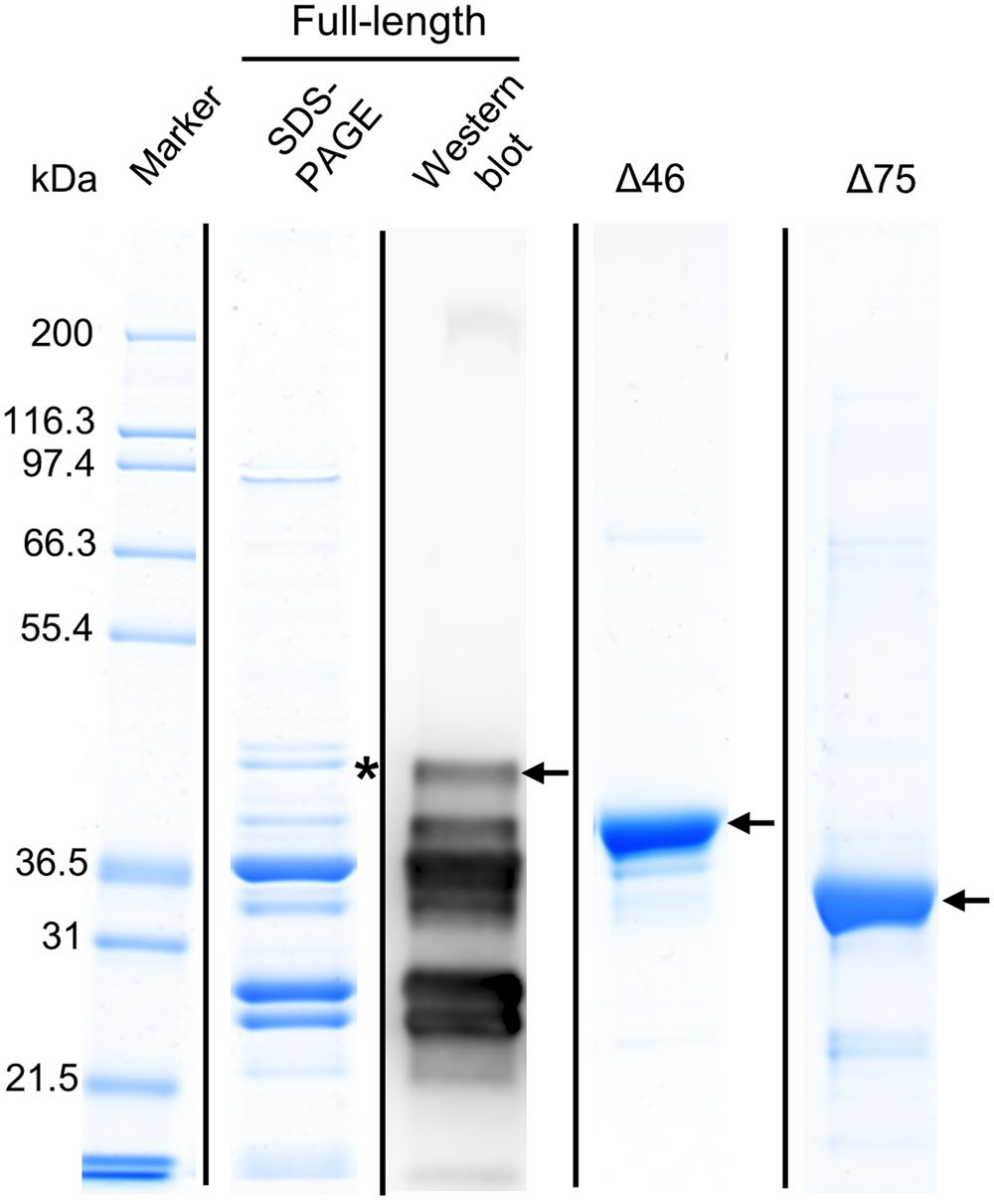
SDS-PAGE and Western-blot analyses of recombinant AtDGAT3 proteins after purification by affinity chromatography. The full-length (40.2 kDa), Δ46 (35.4 kDa) and Δ75 (31.9 kDa) protein variants of recombinant His-tagged AtDGAT3 were purified on a nickel chelating resin from the 12 000 x *g* supernatant of the lysate of an *E. coli* culture and further purified using size exclusion chromatography (Δ46). Protein concentration was determined by the Bio-Rad assay and 7.5 to 10 µg were separated by SDS-PAGE. Each variant protein is indicated by a black arrow. The identity of the band corresponding to the full-length protein was validated by Western blot and by mass spectrometry. The band (highlighted with a black asterisk) of the SDS-PAGE was cut, digested and analysed by LC-MS/MS. Vertical lines indicate delineation of the figure. Original gels and blot are displayed in Supplementary Fig. S2A–C.

AtDGAT3 is predicted to be a soluble protein according to its hydropathy plot (Fig. S1). Accordingly, a transient expression of the Δ75AtDGAT3-GFP fusion in tobacco leaves indicated a cytosolic localization^12^. Full-length AtDGAT3 coding sequence and truncated versions (Δ75 and Δ46) were cloned in the pET32b expression plasmid and corresponding proteins were expressed in fusion with a 6xHis tag localized in C-terminus. When starting from 500 ml of culture, 3 to 6 mg of 98% pure Δ46 or Δ75 proteins were obtained from the cell lysate supernatant on a nickel chelating resin as shown by gel electrophoresis (Fig. 3**)** and a determination of protein concentration. Gel electrophoresis and Western blot analysis showed that the purified full-length protein displayed an extended degradation pattern (Fig. 3). The full-length protein itself appeared as a minor band on the gel, which contained mostly AtDGAT3, as confirmed by mass spectrometry. As a conclusion, it appeared that truncated versions of AtDGAT3 were more stable than the full-length protein. Upon further purification by size-exclusion chromatography, the Δ46AtDGAT3 eluted as a trimer (Fig. S3), assuming it is globular.

### AtDGAT3 is an iron-sulfur protein with a [2Fe-2S] cluster

**Figure 4:**
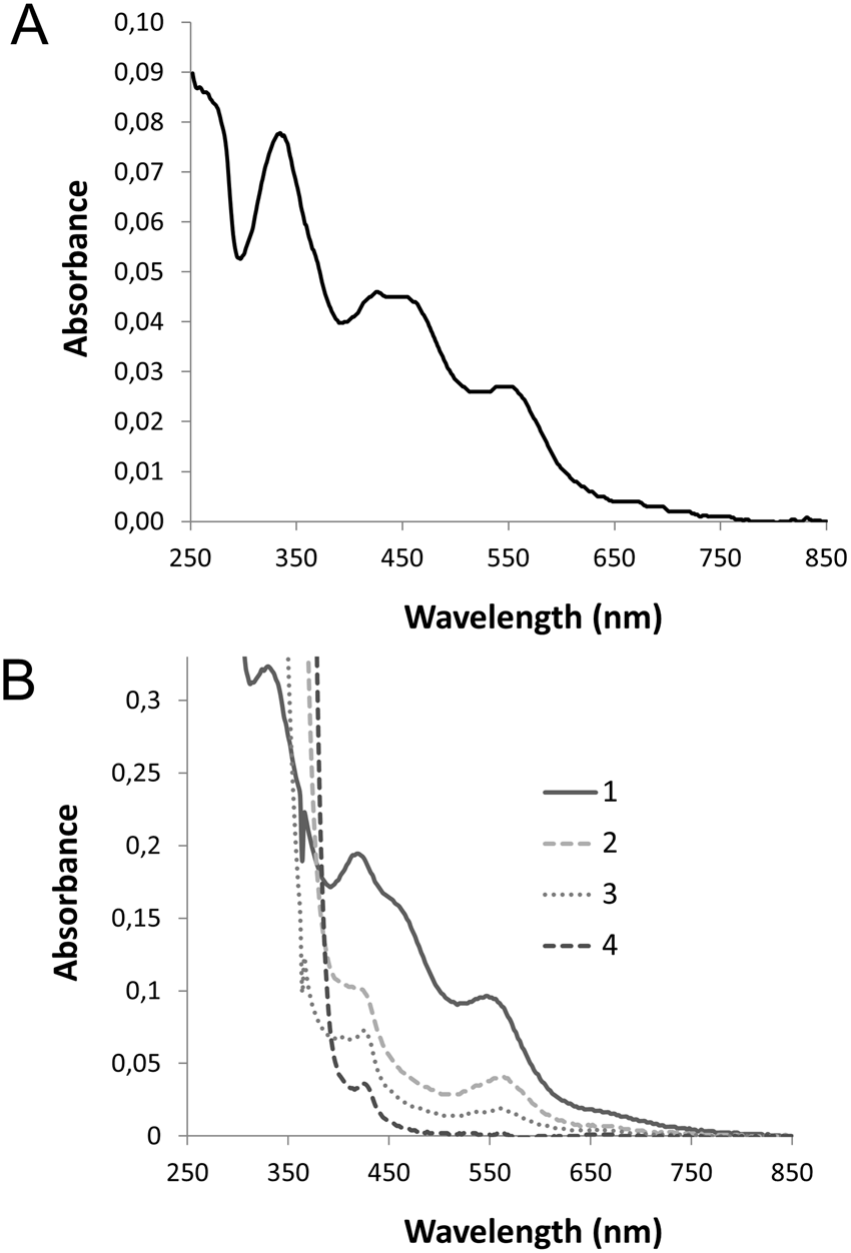
AtDGAT3 UV-visible spectroscopy. (**A**) Freshly purified Δ46AtDGAT3 UV-visible spectrum was registered in aerobic conditions with 16,5 μM of protein on a Shimadzu UV-1800 spectrophotometer. The UV-Vis spectrum presents a typical oxidized [2Fe-2S]^2+^ cluster with sulfur to iron charge transfer absorption bands observed here at 335 nm, 425 nm, and 550 nm. (**B**) Δ46AtDGAT3 UV-visible spectrum of a freshly purified protein at 337 µM was recorded before (spectrum 1) and after addition of sodium dithionite at 2 mM (spectrum 2: 1 minute; spectrum 3: 30 min), or 100 mM (spectrum 4: 20 min).

In the course of AtDGAT3 purification, we observed a brown color associated with the fraction enriched in the protein of interest. Under aerobic conditions, the brown color faded within hours. As sequence alignments (Fig. 1) suggested that the protein could bind a [2Fe-2S] cluster in its thioredoxin-like ferredoxin domain, we performed a biochemical and spectroscopic characterization of the putative iron-sulfur cluster of the protein. We characterized the cluster of Δ46AtDGAT3 because this truncated version of the protein was more stable *in vitro* than the full lenght protein. Iron measurement indicated 1.27 ± 0.04 Fe and 1.55 ± 0.06 S per monomer, so that the purified protein contained around 70% of [2Fe-2S]^2+^ per AtDGAT3 monomer purified under aerobic conditions and immediately stored into liquid nitrogen. The light absorption spectrum of the aerobically purified enzyme displayed a typical [2Fe-2S]^2+^ cluster with sulfur-to-iron charge transfer bands at 335 nm, 425 nm, and 550 nm (Fig. 4A).

Reduction of the Δ46AtDGAT3 protein in the presence of 2 mM sodium dithionite was also monitored by UV-visible spectroscopy. A fast bleaching of the solution as well as a fast decrease of the visible absorption bands were observed (Fig. 4B).

**Figure 5:**
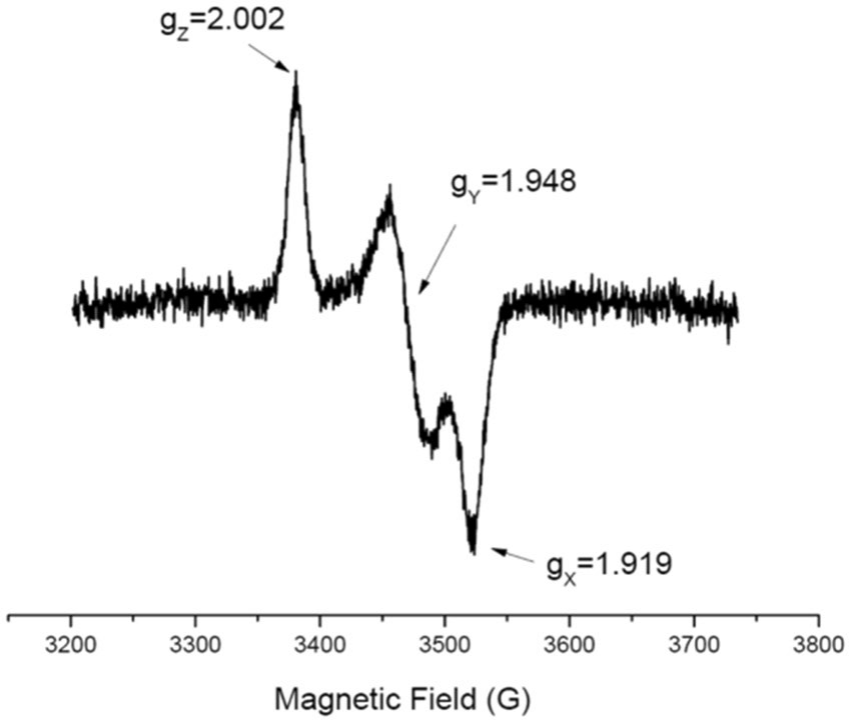
EPR Spectrum of the [2Fe-2S]^+^ cluster of Δ46AtDGAT3. The EPR spectrum of an AtDGAT3 solution (concentration: 250 μM) was recorded at 50 K under non-saturating conditions. The spectrum indicates a rhombic symmetry with 3 distinct principal g-values (g_z_ = 2.002, g_y_ = 1.948, g_x_ = 1.919).

Further characterization of the cluster within AtDGAT3 was carried out by continuous wave EPR. Under microwave power non-saturating conditions at 50 K, AtDGAT3 (either Δ46 or Δ75 truncated version) protein did not display any EPR signal, in agreement with the presence of a S = 0 [2Fe-2S]^2+^ cluster. Figure 5 shows the EPR spectrum of Δ46AtDGAT3 after reduction with 1 mM sodium dithionite. It displays a signal characteristic for a S = 1/2 [2Fe-2S]^+^ cluster with rhombic symmetry *i.e*. with 3 distinct principal g-values (g_z_ = 2.002, g_y_ = 1.948, g_x_ = 1.919). This spectrum accounted for 15.3 µM spins. On the basis of a protein concentration of 250 µM and 70% of [2Fe-2S] cluster per monomer, full reduction would have generated a spin concentration of 175 µM. This result is in line with the loss of iron upon reduction with sodium dithionite and strongly suggests that while AtDGAT3 [2Fe-2S]^2+^ cluster is stable, the reduced EPR active [2Fe-2S]^+^ is rapidly destroyed.

### Recombinant AtDGAT3 has DGAT activity

**Figure 6:**
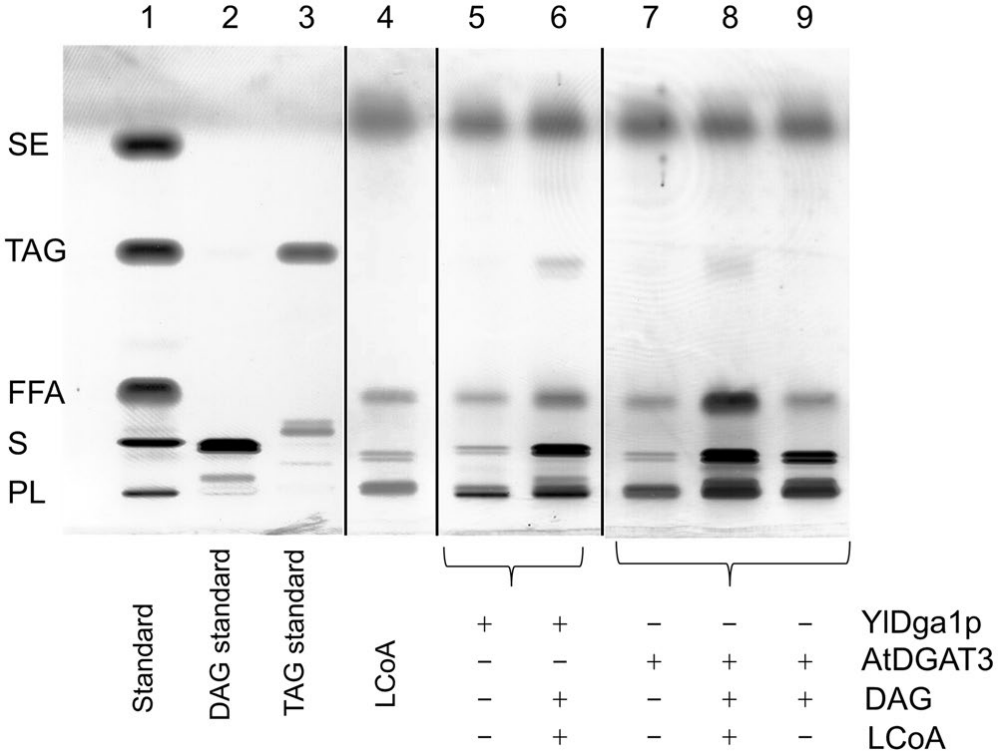
AtDGAT3 catalyses the synthesis of triacylglycerols. HPTLC plate showing the result of DGAT assay using an unlabelled DAG acceptor (1,2-dioleoyl-*sn*-glycerol) and an acyl donor (Lauroyl Coenzyme A). Assays contained 60 µg proteins and were performed during 20 h at 30 °C under mild shaking. Reaction products were extracted and separated on HPTLC plates. Lipid identification was based upon migration obtained for lipid standards. + or − indicate the presence or absence of the different compounds in the reaction mixtures. Vertical lines indicate delineation of the figure. Original HPTLC is displayed in Supplementary Figure S4.

The full-length AtDGAT3 protein purified by immobilized-metal affinity chromatography was used to assay DGAT activity with unmarked DAG as acceptor and linoleoyl-CoA as acyl donor. Upon separation of the substrates and products on HPTLC plate (Fig. 6), we were able to detect significant amounts of TAGs produced by the enzyme. As a control for TAG production, we used the recombinant and purified DGAT2 from *Yarrowia lipolytica* (YlDga1pΔ19)^39^. Upon removal of the 46 or 75 N-terminal residues, the enzyme did not show any TAG synthesis activity in our hands (Fig. S5).

## Discussion

According to public transcriptomic resources, At1g48300 appears to be ubiquitously expressed, with high levels of mRNA accumulation during the late stages of seed development (Arabidopsis eFP Browser, http://bar.utoronto.ca). Accordingly, we could amplify and sequence *AtDGAT3* CDS from a mixture of cDNA prepared from *A. thaliana* maturing seeds. The CDS amplified, which is 1,083-bp long, fits the recently updated gene structure displayed by the TAIR website for At1g48300 and codes for a 360-residues protein corresponding to a calculated MW of 39.2 kDa. AtDGAT3 protein was detected by western blot in germinating seeds 48 h after exposure to the light, just after the emergence of the radicle. The apparent molecular weight of the protein expressed *in vivo* in our experimental conditions is in accordance with that of the full length enzyme. The slightly increased MW of AtDGAT3 observed during the course of seed germination could be due to post-translational modifications of the protein.

When using the full-length AtDGAT3 sequence to query ChloroP 1.1 (http://www.cbs.dtu.dk/services/ChloroP/), a N-terminal chloroplast transit peptide is predicted (46 residues). Using TargetP 1.1 (http://www.cbs.dtu.dk/services/TargetP/), chloroplastic subcellular localization was proposed; however the reliability of the prediction was low (rated 4 among 5 levels, 5 being the less reliable). Saha *et al*.^13^ and Hernandez *et al*.^12^ reported a cytosolic location of AhDGAT3 and AtDGAT3, respectively. However, Saha *et al*.^13^, who performed differential centrifugation to fractionate intracellular compartments from immature *Arachis hypogea* seeds and found active DGAT3 in the cytosolic fraction, also detected DGAT3 activity in other fractions, including the plastidial one. About Hernandez *et al*.^12^, it should be mentioned that the authors expressed a Δ75AtDGAT3 deletion variant lacking the putative chloroplast-targeting signal. Further investigation such as chloroplast import studies are now required to address the question of the subcellular localization of AtDGAT3.

Alignment of DGAT3 plant sequences highlighted different motifs of interest resembling conserved motifs previously described in other acyltransferases (Fig. 1A). Two motifs could be identified by homology in *A. hypogea* DGAT3s only. For the one close to the N-terminus, the HX_4_D motif is shared by members of the glycerol-3-phosphate acyltransferase (GPAT) family^43^, despite a different spacing of histidine and aspartate residues in AhDGAT3s (HX_5_D). Nevertheless, the importance of these motifs has to be taken with caution since *Streptococcus pneumoniae* GPAT, the three dimensional structure of which has been recently resolved^44^, is active despite the lack of a HX_4_D motif. The second motif of interest was identified in *A. hypogea* DGAT3s by homology with members of the DGAT1 family: this motif corresponds to the putative RX_3_EL active site of these enzymes^18,45^. However, multiple sequence alignment shows that these motifs are not conserved among DGAT3s. Their importance for acyltransferase activity should therefore be challenged using site-directed mutagenesis.

So far, a DGAT activity was only demonstrated for two DGAT3 enzymes from *A. hypogea*^11,13^ and the contribution of these proteins to TAG biosynthesis remains to be proven *in planta*. DGAT activity has also been observed for a *Chlamydomonas reinhardtii* enzyme from DGAT3 family expressed in *E. coli*^46^. We used a simple *in vitro* assay for determining the activity of the different forms of bacterially expressed AtDGAT3 corroborating the DGAT activity of this protein.

A careful observation of the time course analysis of purified recombinant AhDGAT3 activity (Fig. 6B in Saha *et al*.^13^) shows that the enzyme first releases free fatty acids (FFAs), and then synthetises TAGs. The results obtained with AtDGAT3 (Fig. 6, lane 8) confirm the capacity of DGAT3 to release FFAs (acyl-CoA hydrolase activity) and demonstrate its ability to synthetize TAGs. Thus, DGAT3s might exhibit an acyl-CoA hydrolase activity.

In order to assess the activity of DGAT3 *in planta*, Hernandez *et al*.^12^ used a truncated DGAT version of the enzyme devoid of TAG synthesis activity in our hands (Fig. S5), maybe due to the loss of the N-terminal GPAT-like motif. They determined the total TAG content and composition of Nicotiana leaves transiently expressing this truncated DGAT3 (Fig. 5 in Hernandez *et al*.^12^) and showed an increase in TAG accumulation with a higher amount of 18:3 and 18:2 FA as compared to a control expressing AtDGAT1. The presence of the [2Fe-2S] cluster in AtDGAT3, the involvement of the enzyme in TAG synthesis and its specificity toward 18:3 and 18:2 FA (Hernandez *et al*.^12^) raises the possibility of a desaturase activity associated with AtDGAT3. One might speculate that Hernandez *et al*.^12^ have observed the result of an acyl-CoA hydrolase activity of the truncated DGAT3 form, associated with a possible reductase activity linked to the [2Fe-2S] cluster. In the present report, the conditions used to assay the activity of the recombinant variants of AtDGAT3 only evidenced an *in vitro* synthesis of TAG with the full-length enzyme. However, an acyl-CoA hydrolase activity could be associated with the deletion variants (Δ75 and Δ46, Fig. S3) and the full-length enzyme. Therefore, one might speculate that the increase in TAG synthesis observed by Hernandez *et al*.^12^ is an indirect consequence of the accumulation of free fatty acid generated by the acyl-CoA hydrolase activity of the cytosolic Δ75AtDGAT3. Together with the acyltransferase activity of another protein, it could explain a slight increase of TAGs enriched in unsaturated FAs.

Little attention has been paid so far to the conserved thioredoxin-like ferredoxin domain located in the C-terminal region of DGAT3s. This domain comprises four conserved cysteines involved in the binding of a [2Fe-2S] cluster, as observed in the crystal structure of previously characterized thioredoxin-like ferredoxin proteins^41,42^. There is some variability in the spacing of the four cysteines. The DGAT3 family presents a spacing (C-X_4_-C-X_27–34_-C-X_3_-C) typical of homologues to this class of ferredoxin^31^. Expression of the At1g48300 gene has previously been linked with iron availability. At1g48300 is overexpressed in leaves and roots during iron deficiency^47^ and downregulated in flowers of a ferritin mutant over-accumulating iron^48^. These transcriptional regulations suggest that the *in planta* function of AtDGAT3 may somehow be linked with iron availability. These observations are in line with the binding of a [2Fe-2S] cluster by the protein, as shown by the biochemical and spectroscopic characterization of the recombinant purified protein presented in this study. To our knowledge, the association of an iron-sulfur cluster with a DGAT enzyme was not been previously reported. This expands the number of functions observed for [2Fe-2S] containing enzymes.

## Electronic supplementary material

Supplementary information
