# Supplementary material for: Arabidopsis thaliana DGAT3 is a [2Fe-2S] protein involved in TAG biosynthesis
Source: Sci Rep. 2018 Nov 22;8:17254. doi: 10.1038/s41598-018-35545-7 (PMC6250708; doi:10.1038/s41598-018-35545-7)
Supplement: Supplementary file 1 — Supplementary information [file 41598_2018_35545_MOESM1_ESM.pdf]

Arabidopsis thaliana DGAT3 is a [2Fe-2S] protein involved in TAG biosynthesis

Laure Aymé[1], Simon Arragain[2], Michel Canonge[1], Sébastien Baud[1], Nadia Touati[3], Ornella Bimai[2], Franjo Jagic[1], Christelle Louis-Mondésir[1], Pierre Briozzo[1], Marc Fontecave[2*], Thierry Chardot[1*]
(1) Institut Jean-Pierre Bourgin, INRA, AgroParisTech, CNRS, Université Paris-Saclay, 78000 Versailles, France
(2) Laboratoire de Chimie des Processus Biologiques, UMR 8229 CNRS, Collège de France, Université Paris 6, 11 Place Marcelin Berthelot, 75231, Paris CEDEX 05, France
(3) Chimie ParisTech, PSL Research University, CNRS, Institut de Recherche de Chimie Paris (IRCP), F-75005 Paris, France.

Correspondence to: thierry.chardot@inra.fr; marc.fontecave@college-de-france.fr

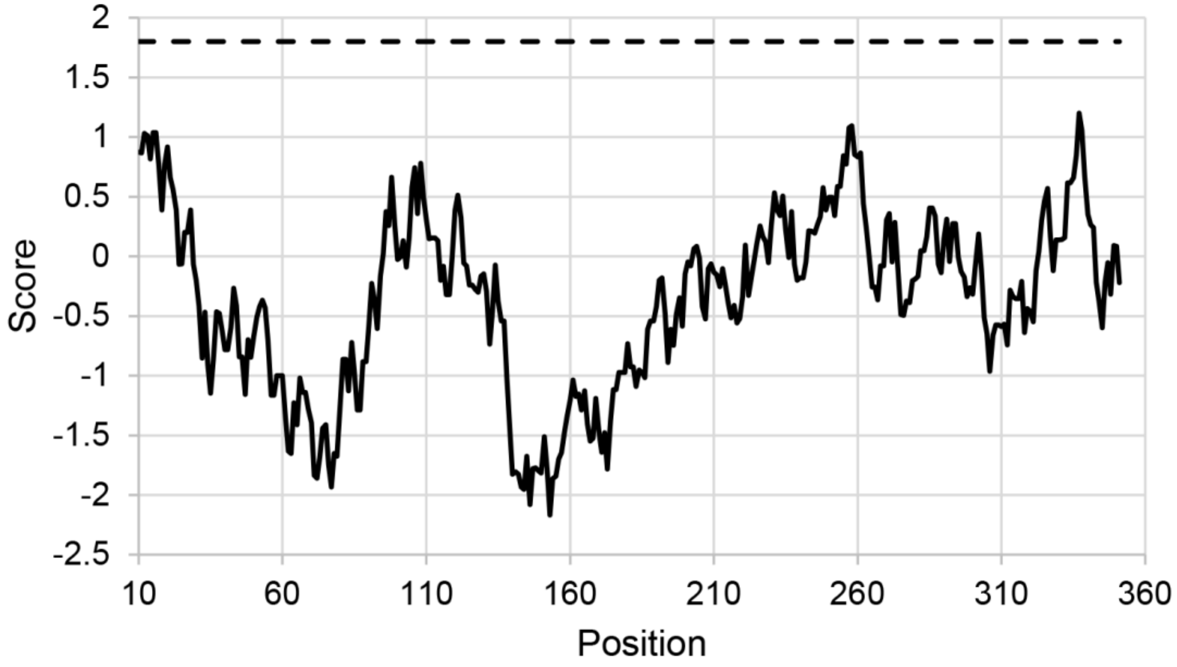

Supplemental Figure S1 : AtDGAT3 Kyte and Doolitle hydropathy plot

The Kyte & Doolittle hydropathy plot of AtDGAT3 sequence was established using the ProtScale tool from the ExPASy portal (http://web.expasy.org/protscale/). The window size corresponds to 19 residues. Hydropathy scores greater than 1.8 (dashed line) indicate possible transmembrane regions. The window position values shown on the horizontal axis reflect the average hydropathy of the entire window, with the corresponding amino acid as the middle element.

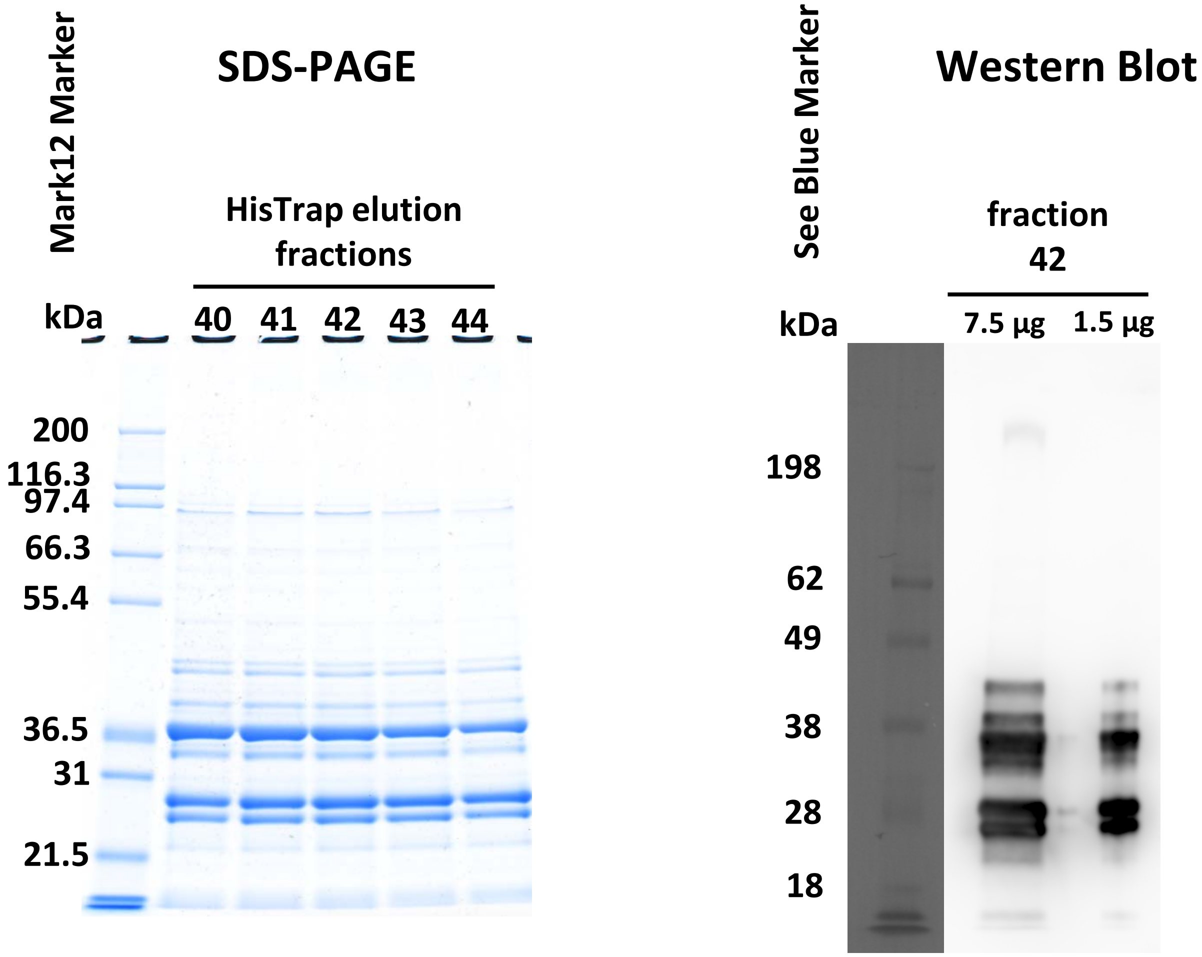

Supplemental Figure S2 A: Purification and immunodetection of full-length AtDGAT3 on HisTrap column

Left panel: Analysis of fraction purity by SDS-PAGE (NuPAGE (ThermoFisher), 15 µg proteins / lane)

Right panel: Western blot analysis of fraction 42 (7.5 and 1.5 µg proteins)

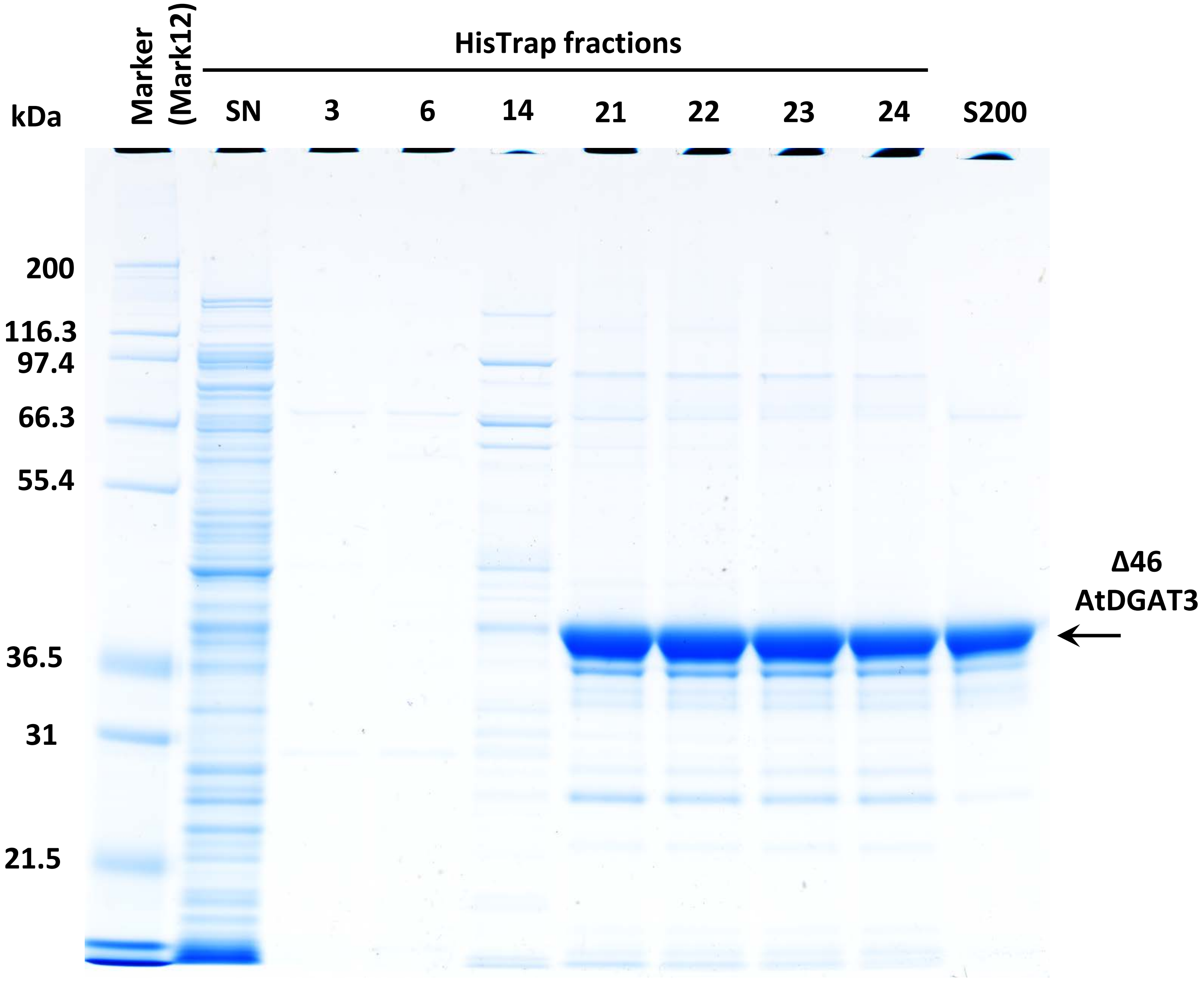

Supplemental Figure S2 B: Purification of Δ46 AtDGAT3

Molecular weight marker Mark 12 (ThermoFisher)

2 µl of the supernatant (SN), 10 µg from fractions 3, 6, 14, 21,22, 23 eluted from HisTrap column were loaded on the gel.

AtDGAT3 purified with HisTrap, was further purified on a Superdex 200 10/300 GL size exclusion column (GE Healthcare) and 10 µg proteins were loaded on the gel (lane S200). This lane was used in figure 3.

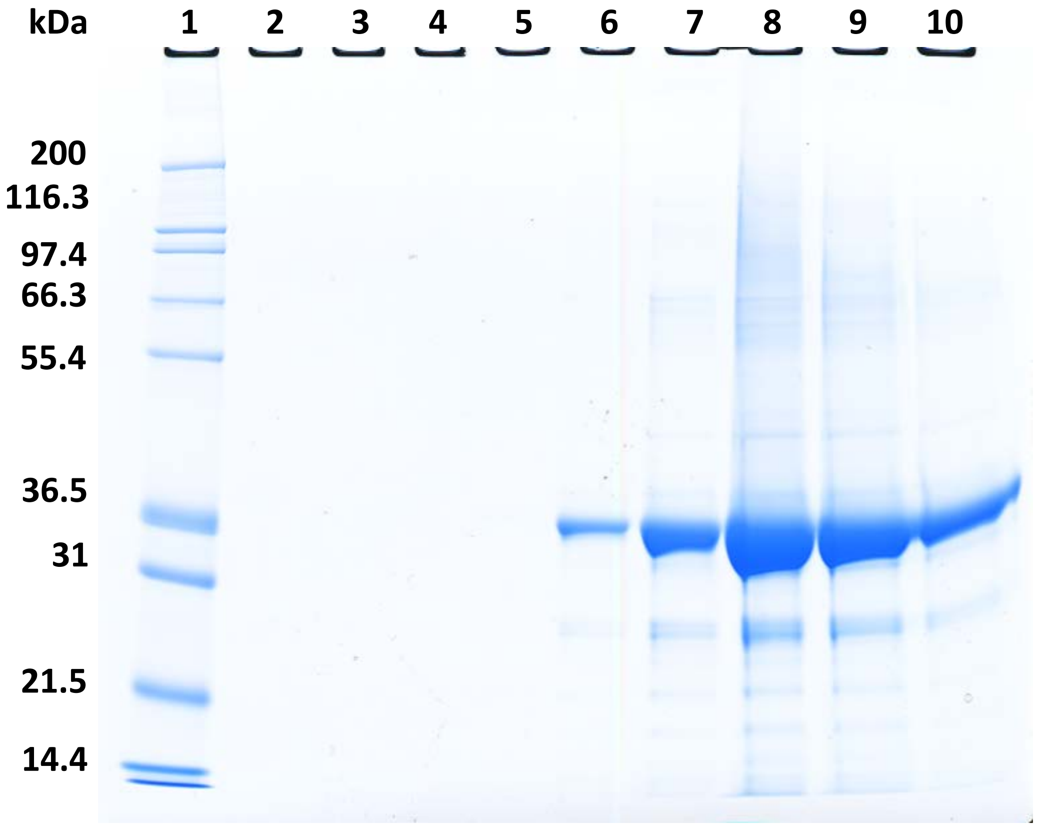

Supplemental Figure S2 C: Purification of Δ75 AtDGAT3
Lane 1: Marker Mark 12 (ThermoFisher)
Lanes 2-5: Flowthrough fractions
HiTrap Q FF ion exchange column (GE Healthcare) was eluted with increasing concentration of NaCl (lanes 6-10)
Fraction 7, eluted with 500 mM NaCl, was shown in figure 3

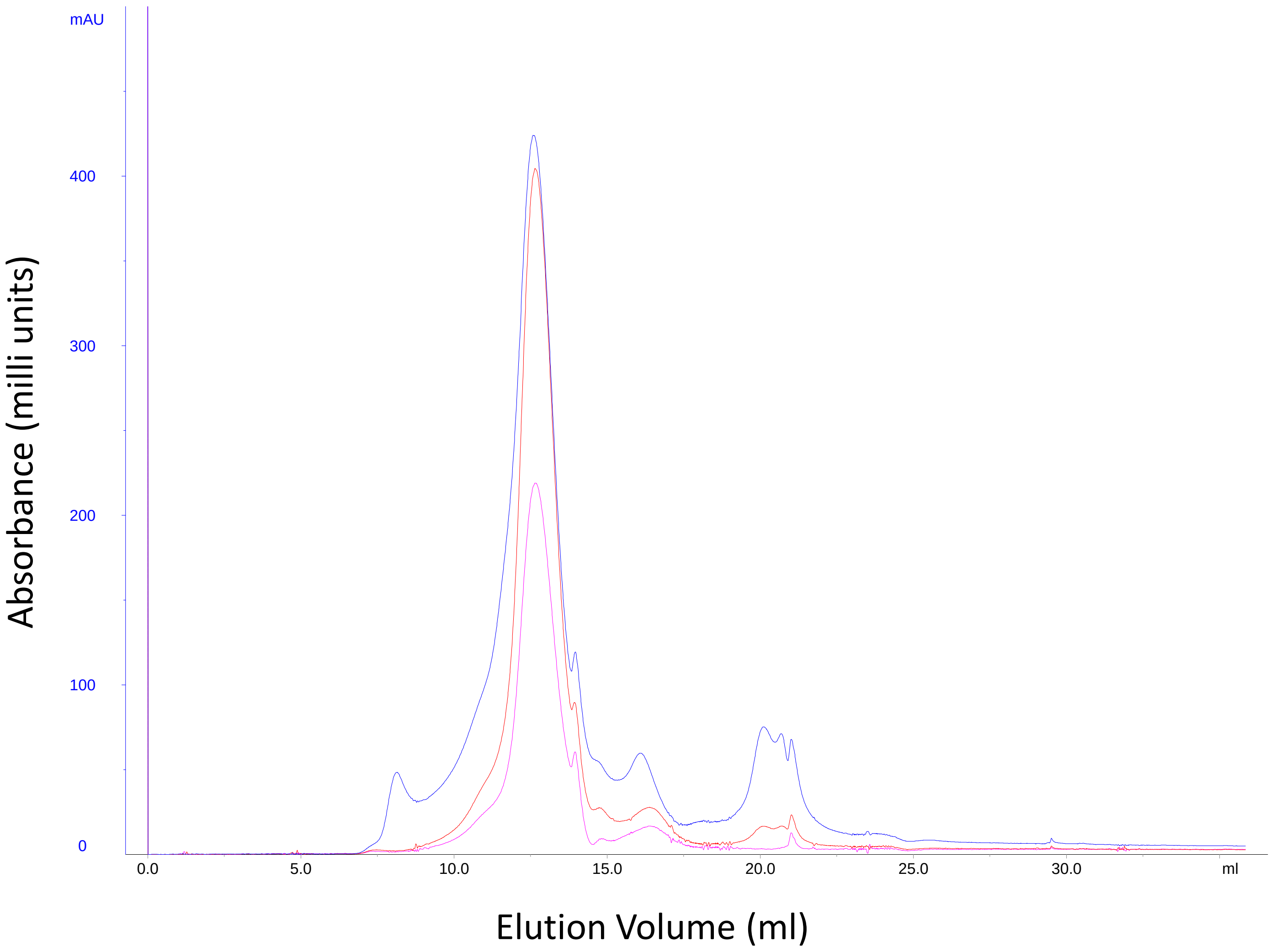

Supplemental Figure S3 : Determination of the oligomerization state of AtDGAT3 by size exclusion chromatography.

Δ46AtDGAT3 (calculated MW of the monomer 35.4 kDa) was separated by size exclusion chromatography on a Superdex 200 column. The absorbance at 280 nm (blue line) was recorded to follow protein elution and the absorbances at 335nm (red line) and 410 nm (magenta line) were used to highlight the presence of the iron-sulfur cluster. The protein eluted in one main peak with an elution volume of 12.6 ml corresponding to a trimer (110 kDa), according to the calibration curve obtained with gel filtration standards separated in the same buffer.

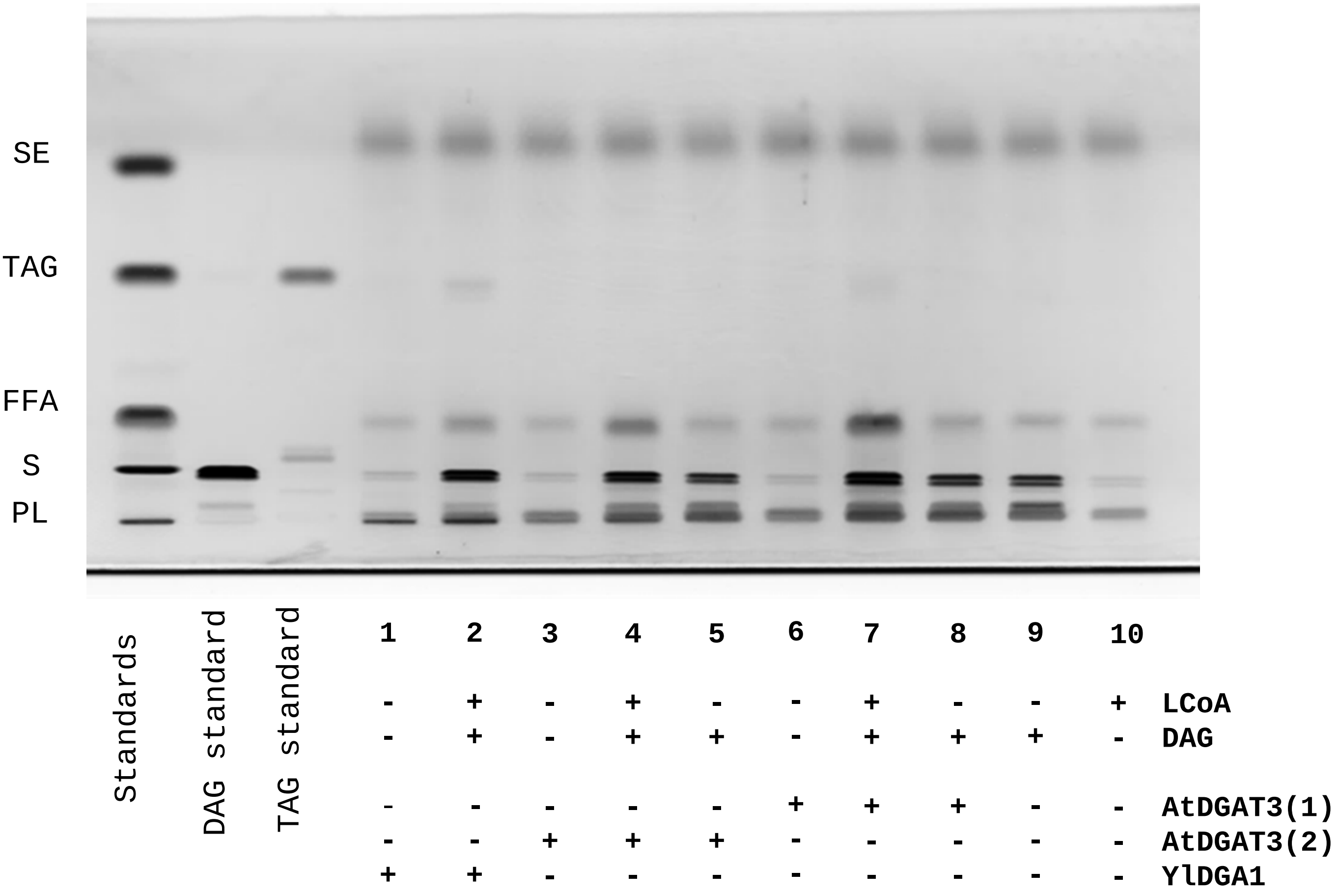

Supplemental Figure S4: HPTLC plate showing AtDGAT3 assays.
Assays contained an unlabelled DAG acceptor (1,2-dioleoyl-*sn*-glycerol) , and LCoA (C18:2-CoA) as an acyl donor for AtDGAT3 and for DGA1. AtDGAT3 (1) and AtDGAT3 (2) correspond to two different fractions of purified recombinant enzyme. AtDGAT3 (1), freshly purified, and immediately assayed, exhibited activity. AtDGAT3 (2) corresponds to a fraction purified similarly to AtDGAT3 (1) but assayed 4.5 h later, and showing no detectable DGAT activity (AtDGAT3 is very sensitive to proteolysis and oxidation). Therefore, results from lanes 6, 7, 8 (using AtDGAT3 (1)) and proper controls were selected in figure 6 (lanes 7, 8, 9) to illustrate the enzyme capacity to catalyse TAG synthesis. Lipid standards : mixture of phospholipids (PL), sterol (S), free fatty acids (FFA), triacylglycerols (TAG) and steryl esters (SE), 2 µg each). DAG (1,2-dioleoyl-*sn*-glycerol) :11.6 µg.

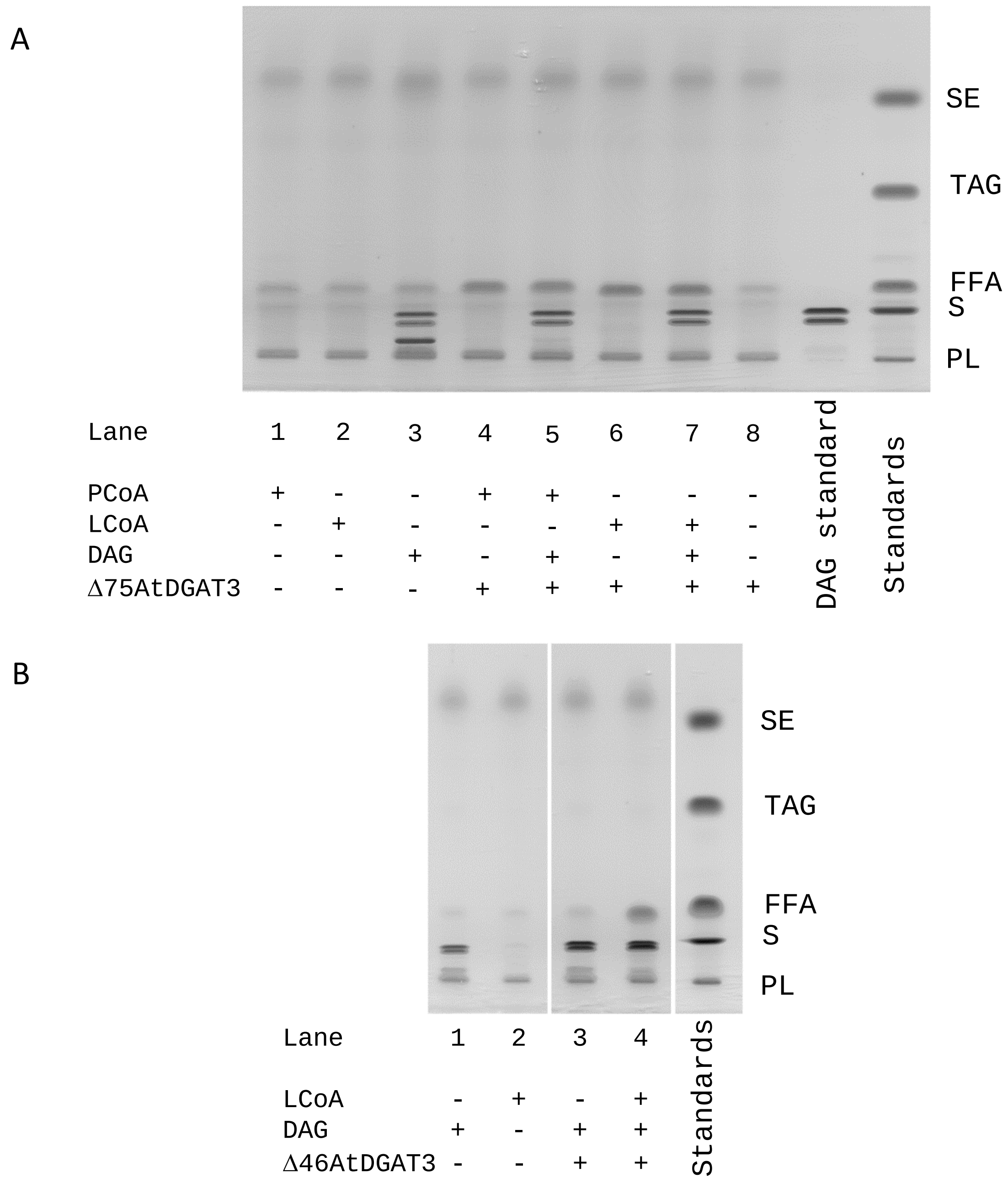

Supplemental Figure S5: HPTLC plates showing AtDGAT3 Δ75 and Δ46 activity assays.

A) LCoA (C18:2-CoA) or PCoA (C18:0-CoA) was used as an acyl donor for Δ75AtDGAT3.

B) LCoA was used for Δ46AtDGAT3 assay.

Assays contained 20 µg (A) or 60 µg (B) of proteins and were performed during 20 h at 30°C under mild shaking. + or – indicates the presence or absence of the corresponding compounds in the reaction mixtures. No formation of TAG was observed. Lipid standards : mixture of phospholipids (PL), sterols (S), free fatty acids (FFA), triacylglycerols (TAG) and steryl esters (SE), 2 µg each. DAG (1,2-dioleoyl-*sn*-glycerol): 11.6 µg.
